# Hepatitis B virus: Prevalence, vaccination coverage and immune responses to immunization among healthcare workers at Muhimbili National Hospital

**DOI:** 10.1371/journal.pone.0321623

**Published:** 2025-04-16

**Authors:** Marythereza Orotta, Patricia Munseri, Regan Valerian Massawe, Godfrey Michael Orotta, Ashabilan Ebrahim, Eka Patricia Kisali, Stanley Kajaja, Brendan Shayo, Amunga Meda

**Affiliations:** 1 Department of Internal Medicine, Muhimbili University of Health and Allied Sciences, Dar es Salaam, Tanzania; 2 Department of Internal Medicine, Muhimbili National Hospital, Dar es Salaam, Tanzania; 3 Department of Physiology, Muhimbili University of Health and Allied Sciences, Dar es Salaam, Tanzania; Al-Azhar University, EGYPT

## Abstract

**Background:**

Healthcare workers (HCWs) are at an increased risk of contracting and transmitting the hepatitis B virus (HBV). Vaccination coverage against HBV a cost- effective prevention, remains low among HCWs in some settings.

**Objective:**

To determine the prevalence of HBV infection, vaccination coverage, and immune responses to HBV vaccine among HCWs at Muhimbili National Hospital (MNH) in Dar es Salaam.

**Methodology:**

This cross-sectional study used a proportional probability systematic sampling to recruit HCWs from MNH in Dar es Salaam. A structured questionnaire was used to collect social demographic characteristics, infection prevention and control practices, HBV vaccination status and reasons for not vaccinating. Five mLs of a peripheral venous sample was obtained from each participant, the sample was used to test for HBV surface antigen, HBV surface antibody and HBV core antibody for assessing for infection, and vaccine immunity respectively. A Robust Poisson Regression analysis was used to assess factors associated with not vaccinating.

**Results:**

The prevalence of HBV infection was 5 (1.2%) among the 415 recruited HCWs. Only 192 (46.3%) HCWs were vaccinated and 169 (96%) had protective immunity against HBV infection among 176 participants who had received at least two vaccine doses. HCWs who were laboratory scientist aPR = 2.01, 95% CI (1.35–3.00) and had < 10 years of employment aPR = 1.62, 95% CI (1.04–2.50) were unlikely to vaccinate against HBV. Vaccine unavailability 114 (51.1%), hesitancy 42 (18.8%), time constraints 41 (18.4%) and financial constraints 26 (11.7%) were factors associated with not vaccinating.

**Conclusion:**

Vaccine coverage against HBV among HCWs at MNH is alarmingly low. Vaccine access, subsidizing cost, protecting time for vaccination could improve vaccination uptake. Qualitative studies are needed to assess for reasons for vaccine hesitancy among HCWs.

## Introduction

Hepatitis B virus (HBV) infection remains one of the major global health concerns with about 296 million people living with the virus and about 820,000 lives claimed from liver cirrhosis and hepatocellular carcinoma in 2019 [1]. The Western Pacific and African regions are the most affected, harboring about 116 million and 81 million people with the infection respectively [1]. Tanzania is among the high-burden countries, with a prevalence ranging between 3.8% to 8% [2] Vaccination against HBV is known to be an effective measure in the prevention for HBV and provides 90–95% protection [1]. However, HBV vaccination coverage remains low ranging from 18–39% in developing countries and 67–79% in developed countries [3]. Health care workers (HCWs) are at increased risk of contracting and transmitting HBV from and to their patients [3,4]. HCWs are expected to have access to screening, prevention, diagnosis, and treatment services for HBV however, in Tanzania it was observed that only 11.3% of HCWs were aware of HBV infection status and 33% were vaccinated [5]. Protective immunity from HBV vaccination is achieved in 90–95% [1] although there have been reports of up to 30% of vaccinated HCWs in Mwanza who had not developed immunity [6]. The lack of immunity in vaccinated HCWs poses a risk of contracting and transmitting HBV infection therefore, it is prudent to screen immunized HCWs [7]. There have been ongoing campaigns at MNH to address HBV prevention and immunization after the last finding in the year 2017 [8]. We aimed to determine the prevalence of HBV infection, HBV coverage and responses to immunization among HCWs at MNH following the campaigns.

## Methodology

### Study design and population

This hospital-based cross-sectional study was conducted among HCWs at Muhimbili National Hospital (MNH) which is a national referral hospital located in Dar es Salaam Tanzania with a bed capacity of 1,500 beds and 2,700 employees. Ethical clearance was obtained from the Research and Ethics Committee of Muhimbili University of Health and Allied Sciences (MUHAS) with ethical approval number MUHAS-REC-08-2023-1859 prior to the study onset. Written informed consent was obtained from each participant. Participants were recruited from 6 September 2023–23 February 2024. Participants were recruited using a probability proportional sampling to select participants within the chosen departments: laboratory, cleaning, dialysis, emergency medicine, surgical and medical wards. The departments were chosen based on risk of HBV exposure among the staff due to their direct and indirect contact with patients, biological samples, and medical equipment.

### Data collection

A structured interviewer-based questionnaire was used to collect study information from study participants. Information collected included: social demographic characteristics, infection prevention and control practices HBV vaccination status, and reasons for not vaccinating among participants that have not been vaccinated. The results for point of care HBsAg rapid test results, HBsAb quantitative and HBcAb qualitative test results of only vaccinated participants were transcribed in the structured questionnaire before data entry.

### Vaccination status and coverage assessment

Vaccination status was assessed for all study participants. Participants with at least two HBV doses within six months to 30 years were assessed for immune responses. Un-vaccinated participants were assessed for reasons for not vaccinating and were thereafter referred to the HBV clinic for vaccination. Participants that were vaccinated were assessed for vaccination details: type of vaccination, number of vaccine doses, adherence to the vaccine schedule, and reasons for non-adherence to the vaccine schedule.

### Hepatitis B infection testing

Five mLs of a venous sample was obtained from each participant to test for HBsAg using a rapid hepatitis B surface antigen diagnostic test Healgen manufactured by Zhejiang Orient Gene Biotech Co., Ltd. Two drops each of fifty microliters of blood and one drop of the buffer were applied to the test strip. After fifteen minutes the test was regarded as Positive if two distinct red lines appeared in the control and test region respectively and negative if only one red line appeared in the control region. The test was regarded as invalid if the control line failed to appear and the test was repeated with a new strip. The sensitivity of the Healgen rapid diagnostic test is 99.4% and the specificity is 99.5%.

### Immune response evaluation and criteria

Immune response to HBV vaccine was ascertained by detecting HBsAB titers of ≥ 10 mIU/mL and negative HBcAb. HBcAb qualitative testing was done by adding 50mls of control to control well, and 50mls of participants samples to assigned wells. Then, 50mls of horseradish peroxidase (conjugated monoclonal HBcAb) was added to each well except blank.

The plate was incubated for 30 minutes at 37°C in a BINDER incubator with the serial number 200285. The competitive principle was used for incubation, where the samples with positive HBcAb competed with horseradish peroxidase for a fixed amount of purified HBcAg pre-coated in the wells. In a sample with negative HBcAb, the horseradish peroxidase was bound directly to antigens inside the wells. To avoid cross-contamination, the wells were washed five times with diluted wash buffer using an automatic well washer.

After washing fifty microlitres of urea peroxide solution and fifty microlitres of Tetramethyl benzene were added to each well. The plate was incubated again for 10 minutes at 37°C.To stop the reaction, a fifty microlitre of diluted 0.5M sulphuric acid was added into each well and mixed gently. In the absence of HBcAb in the sample, a blue color appeared that turned yellow with sulphuric acid. The presence of HBcAb in the sample was indicated by low color or no color. The plate reader with serial number 357-90076z8 was calibrated with absorbance at 450nm and the reference wavelength was set at 630nm within ten minutes after stopping the reaction. Results were calculated by relating each sample absorbance to the cut-off value (sum of positive control times 0.6 and negative control times o.4) of the plate. The result was negative if the specimen was giving absorbance more than the cut-off value and positive if lower than the cutoff value. Test results were valid if quality control criteria were fulfilled; The blank well had to be< 0.080 at 450nm, the negative control ≥ 0.80 at 450/630nm, and the positive control < 0.100 at 450/630nm. Each microplate was evaluated independently for quality control.

The sandwich ELISA method was used for HBsAb quantification, fifty microlitres of the sample were added into the recombinant HBsAg precoated wells followed by fifty microlitres of conjugated Horseradish peroxidase. The plate was covered and incubated for 60 minutes at 37°C. If the sample contained HBsAb, the antigen-antibody immunocomplex was formed and captured on the solid phase during incubation. Each well was washed five times with buffer by Thermoscientific well wash with the serial number N10800-0519111551 to remove unbound HRP conjugates and serum proteins preventing false positives and high background. Fifty microlitres each of urea peroxidase and tetramethyl benzadine solution were added into each well and incubated for 10 minutes at 37°C avoiding light. After adding fifty microlitre of 0.5M sulphuric acid to stop the blue-colored antigen-antibody complex reaction, the blue color turned yellow, with intensity proportional to the amount of antibody in the sample, indicating a positive HBsAb result. Wells remained colorless if HBsAb was negative. The plate reader was calibrated at 450nm and the reference at 630nm. The absorbance values were used to determine HBsAb concentration from the standard curve. For quality control, the dose-response curve must meet set parameters, four out of six control calibrators should fall within established ranges. ELISA KIT had a sensitivity of 98% and a specificity of 99%.

### Data analysis

Data was collected with REDCap version 11.1.5 and exported to Microsoft Excel 2016 for cleaning and transferred into STATA version 15.1 for analysis. Frequency tables and percentages were used to summarize categorical variables. Continuous variables were summarized as median and interquartile ranges. The prevalence of HBV was calculated as the total number of participants with a positive HBsAg divided by all tested participants. The proportion of participants vaccinated was computed by dividing the total number of vaccinated participants by all recruited participants. Immune response was calculated by dividing the total number of participants with HBsAbs titers ≥ 10 mIU/mL to all vaccinated participants. A comparison of proportions was done by using Chi-square test.

Robust Poisson regression was used to assess for factors associated with not vaccinating. Factors with a p-value <0.2 in the bivariable model were included in the multivariable Poisson regression model after controlling for confounders and effect modifiers a p-value <0.05 in the multivariate model was considered statistically significant.

## Results

### Recruitment and participation in HBV testing, vaccination status assessment and immunity to HBV among study participants

**Fig 1:**
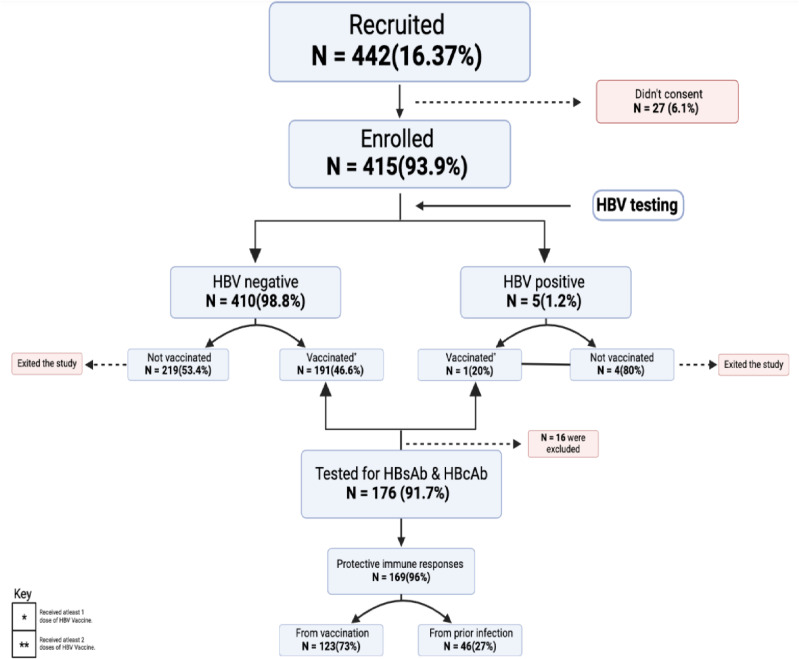
A study consort diagram showing the recruitment and flow of study participants. *Received at least one dose of HBV Vaccine. **Received at least two doses of HBV Vaccine.

The consort diagram “Fig 1” summarizes the recruitment and flow of study participants, 442 (16.37%) HCWs from six departments were approached 415 (93.90%) participants were enrolled 27(6.10%) refused to consent. All 415 (100%) recruited participants were tested for HBV infection and then assessed for vaccination status. We assessed immune response in 176 (91.67%) participants who received at least two vaccine doses in not less than six months and not more than 30 years.

### Social-demographic and behavioral characteristics of study participants

Table 1 summarizes the socio-demographic and behavioral characteristics, in total 415 participants were recruited, 272 (65.5%) were female, the median age was 31.39, IQR [26.64–41.02] years and median employment duration of 3 IQR [0.92–10.79] years. Among the study participants; 187 (45%) had attained certificate/ diploma education, 264 (63.6%) were nurses and 206 (49.6%) were from Surgical wards. The majority 330 (79.5%) had infection prevention and control training, 399 (96.1%) reported having an SOP in their working area and 399 (100%) reported adhering to the SOP. A total of 252 (60.7%) reported exposure to patients’ body fluids, 412 (99.3%) reported to having (Personal protective equipment) PPE access while 162 (39%) reported reusing PPE.

**Table 1:** Social-demographic and behavioral characteristics of study participants (N=415).

Variable	N=415	Percent (%)
Median Age (years)	31.39	[IQR] 26.64–41.0 3
Median Employment duration (Years)	3	[IQR] 0.92–10.79
Female	272	65.5
**Highest Education level attained**		
Primary school	30	7.2
Secondary school	24	5.8
Certificate/ diploma	187	45.0
University and higher	174	41.9
**Profession**		
Cleaner	44	10.6
Health laboratory professional	27	6.5
Nurse	264	63.6
Doctor	80	19.3
**Working department**		
Cleaning	44	10.6
Dialysis	18	4.3
Emergency medicine	23	5.5
Laboratory	27	6.5
Medical wards^*^	97	23.4
Surgical wards^*^	206	49.6
**IPC behavior**		
No IPC training	85	20.5
Exposure to patient body fluid	252	60.7
SOP unavailable in the work area	16	3.9
PPE Access	415	100
Does not use PPE	3	0.7
PPE reuse	162	39

*Medical wards represent Internal Medicine, and Pediatrics Surgical wards represent Surgery, Dental, Gynecology and Obstetrics and theatre

### Prevalence of Hepatitis B virus infection among study participants

The prevalence of HBV infection among the study participants was 5 (1.2%); 1 cleaner, 1 doctor, and 3 nurses. None of the HBV-infected participants met the criteria for antiviral therapy. One participant with HBV infection had received three doses of HBV vaccine and adhered to schedule

### Social-demographic characteristics of vaccinated and unvaccinated participants

A total of 192 (46.3%) HBV vaccinated participants, 16 (8.3%) received only a single dose, 49 (25.5%) received two doses, 119 (62%) received three doses and 8 (4.2%) received three doses with an additional booster dose.

**Table 2** summarizes the social demographic characteristics of the vaccinated and unvaccinated participants. The median age of vaccinated participants was 35.2 years IQR [28.8–46.4] years and was 29.0 years IQR [25.8–35.9] years among the unvaccinated participants p values <0.001.

**Table 2:** The social demographic characteristics of the vaccinated and unvaccinated participants (N=415).

Characteristics	Vaccinated participants N=192 (46.3%)	Unvaccinated participants N=223 (53.7%)	*p*-value
Median age in years (**IQR**)	35.2 (28.8-46.4)	29.0 (25.8-35.9)	<0.001
Duration of employment in years			<0.001
<=10	118 (38.31)	190 (61.69)	
>10	74 (69.16)	33 (30.84)	
**Sex**			0.285
Female	131 (48.2)	141 (51.8)	
**Highest Education level attained**			<0.001
Primary school	8 (26.7)	22 (73.3)	
Secondary school	0 (0)	24 (100.0)	
Certificate/ diploma	89 (47.6)	98 (51.4)	
University and higher	95 (54.6)	79 (45.4)	
**Profession**			<0.001
Cleaner	1 (2.3)	43 (97.7)	
Health laboratory professional	6 (22.2)	21 (77.8)	
Nurse	133 (50.4)	131 (49.6)	
Doctor	52 (65.0)	28 (35.0)	
**Working department**			<0.001
Cleaning	1 (2.3)	43 (97.7)	
Dialysis	7 (38.9)	11 (61.1)	
Emergency medicine	13 (56.5)	10 (43.5)	
Laboratory	6 (22.2)	21 (77.8)	
Medical wards^*^	44 (45.4)	53 (54.6)	
Surgical wards^*^	121 (58.7)	85 (41.3)	
**IPC Parameter**			
No IPC training	35 (41.2)	50(58.82)	0.291
Exposure to patient body fluid	139 (55.2)	113 (44.8)	<0.001
SOP unavailable in the work area	7 (43.8)	9 (56.3)	0.837
PPE Access	192 (46.27)	223 (53.7)	0.528
Does not use PPE	1 (33.3)	2 (66.7)	0.652
PPE reuse	66 (40.7)	96 (59.3)	0.071

*Medical wards represent Internal Medicine, and Pediatrics. Surgical wards represent Surgery, Dental, Gynecology and Obstetrics and theatre

Only 118 (38.3%) participants with 10 or less years of employment had vaccinated compared to 74 (69.16%) with over 10 years of employment p value <0.001. Only 1 (2.3%) cleaner, 6 (22%) of the laboratory workers were vaccinated while vaccination rates for doctors was 52 (65%), and nurses 133 (50.4%) p value <0.001. There were 139 (55.2%) participants who reported to be exposed to patient body fluids in the group that was vaccinated p value <0.001. A total of with 35 (41.2%) participants that did not receive IPC training had vaccinated.

### Participant’s reasons for not vaccinating against HBV

**Fig 2:**
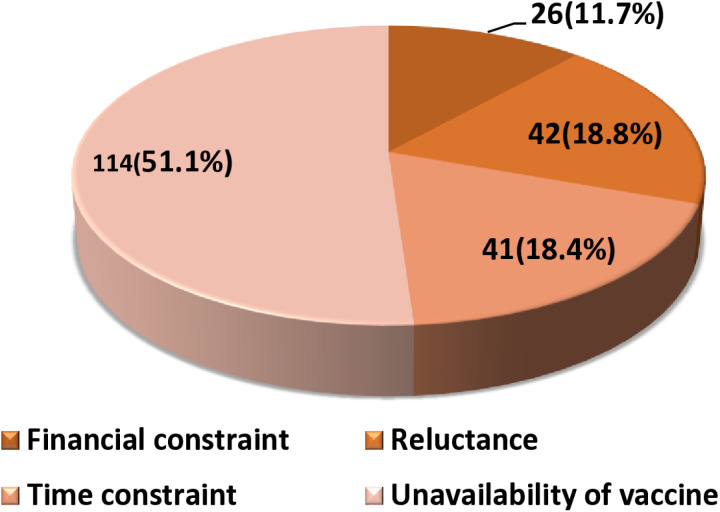
Study participants’ reasons for not vaccinating (N = 223).

Participant’s reasons for not vaccinating against HBV are summarized in “Fig 2”, of the 223 (53.7%) participants who had not received a single HBV vaccine dose, 114 (51.1%) reported that they had not been vaccinated because the vaccine was not available, 42 (18.8%) were reluctant to receive the vaccine, 41 (18.4%) indicated that they had no time and 26 (11.7%) indicated that they were financially constrained.

### Factors associated with not vaccinating among study participants

In a bivariable logistic regression analysis Table 3, factors associated with not vaccinating included: age group of 19–24 years {PR 2.64, 95% CI (1.55–4.49)}, <=10 years employment {PR 2.00, 95% CI (1.49–2.69)}, lower education levels of primary {PR 1.61, 95% CI (1.23–2.12)}, and secondary school {PR 2.20, 95% CI (1.87–2.59)}, Cleaner’s {PR 2.79, 95% CI (2.06–3.78)}, Laboratory workers {PR 2.22, 95% CI (1.55–3.19)}, and nurses {PR 1.42, 95% CI (1.03–1.96)}.

**Table 3:** Factors associated with not vaccinating for HBV among study participants (N=223).

Variable	Bivariable Robust Poisson Regression		Multivariable Robust Poisson Regression	
**PR (95%CI)**	*p* **-value**	**aPR (95%CI)**	*p* **-value**
*Age groups (years)*				
19–24	2.64 (1.55−4.49)	<0.001	1.26 (0.61–2.57)	0. 533
25–34	1.84 (1.09−3.11)	0.022	1.13 (0.56–2.28)	0.725
35–44	1.39 (0.79−2.44)	0.251	1.01 (0.51–2.01)	0.98
45–54	1.08 (0.56−2.06)	0.819	0.91(0.47–1.78)	0.787
>54	Ref		Ref	
*Duration of employment (years)*				
<=10 years	2.00 (1.49−2.69)	<0.001	1.62 (1.04–2.50)	0.031
> 10 years	Ref		Ref	
*Education level*				
Primary school	1.61(1.23−2.12)	0.001	1.11(0.39–3.17)	0.852
Secondary school	2.20(1.87−2.59)	<0.001	1.07(0.37–3.10)	0.899
Certificate/ diploma	1.15 (0.93−1.43)	0.187	1.19(0.95–1.51)	0.129
University and others	Ref		Ref	
**Profession**				
Cleaner	2.79 (2.06−3.78)	<0.001	2.56(0.86–7.51)	0.092
Health laboratory scientist	2.22 (1.55−3.19)	<0.001	2.01(1.35–3.00)	0.001
Nurse	1.42 (1.03−1.96)	0.034	1.40 (0.97–2.03)	0.076
Doctor	Ref		Ref	
***Department***				
Cleaning	2.36 (1.99−2.80)	**<**0.001	Omitted	
Dialysis	1.48 (0.99−2.21)	0.056	1.13 (0.74–1.70)	0.572
Emergency medicine	1.05 (0.64−1.73)	0.836	1.02 (0.65–1.62)	0.919
Laboratory	1.88 (1.45−2.44)	**<**0.001	Omitted	
Medical wards	1.32 (1.03−1.69)	0.024	1.24 (0.98–1.58)	0.069
Surgical wards	Ref		Ref	

However, in multivariable logistic regression analysis Table 3, only employment <= 10 years {aPR 1.62 95% CI (1.04–2.50)} and laboratory workers {aPR 2.01 95% CI (1.35–3.00)} were significantly associated with not vaccinating.

### Protective immune response from HBV vaccination among study participants (N=176)

Immune response was evaluated in 176 participants who received at least two vaccine doses within six months and thirty years duration. Of the 176 participants, 169 (96%) had mounted protective HB surface antibody titer levels against HBV infection. Among 176 participants, 46 (26.1%) had protective immunity from previous infection and 123(69.9%) had protective immunity from vaccine. Among 7 participants that did not mount adequate antibody titers to confer immunity 5 (71.4%) participants had three immunization doses according to the vaccine schedule.

## Discussion

Healthcare workers face an increased risk of contracting HBV infection compared to the general population as a result of exposure through direct contact with HBV-infected blood and body fluids from patients [9]. The prevalence of HBV infection among HCWs at MNH in this study was 1.2% which is lower compared to other settings such as 8.1% in Mulago Tertiary and Teaching Hospital in Uganda in 2010 and 10.6% in Nigeria in 2019, 18.9% in Congo in 2015, 7% in Bugando Medical Center Tanzania in 2012 [6,10–13]. The low observed prevalence in a setting with low vaccine coverage could be attributed to adherence to infection prevention and control practices, or from acquired immunity from previous HBV infection. Though in this study we were unable to establish the timing for previous HBV infection. Consequently, it is possible that the infections occurred long before implementing the current infection control measures. Over the years, enhancements in public health strategies, increased awareness, and better implementation of preventive measures may have contributed to a reduction in HBV prevalence among HCWs in some areas.

This comparison from the past emphasizes the importance of continuous monitoring and adaptation of HBV prevention strategies. It serves as a reminder that healthcare facilities must remain vigilant and proactive in their efforts to control HBV infection rates, as past experiences demonstrate how quickly these rates can escalate if preventive measures are not adequately maintained and updated. While the low prevalence of HBV infection among HCWs at MNH is encouraging from a public health standpoint, it is essential to avoid complacency. Continued vigilance and proactive measures are necessary to sustain this positive trend and protect healthcare workers and patients from potential infection. A prevalence of 1.2% among HCWs poses a risk, for transmission to patients, highlighting the need for continuous surveillance and adherence to infection prevention and control practices, coupled with vaccination campaigns among HCWs.

The HBV immunization coverage among HCWs at MNH is alarming low with only 46.3% of the HCW vaccinated. This figure falls significantly short of the WHO recommendations that advocate for vaccination of all high-risk populations including HCWs (83). The low HBV vaccination coverage among HCWs in Africa is of significant concern, with reported overall rates as low as 18% [14]. HBV vaccination coverage among HCWs in Nigeria was 35% [12] in Ethiopia was 12.9% HCWs and in Rwanda was only 4.5% [12,15,16]. These figures highlight the urgent need for improved vaccination programs and initiatives aimed at protecting HCWs across the continent. The reasons for low vaccination coverage in our study were limited access and high cost of HBV vaccine, while individual factors were lack of time and hesitancy. In some regions such as Brazil and Turkey vaccine coverage was at 95% and 88.2% respectively [17,18]. Improved vaccine access and availability of the vaccine at a nominal fee or the hospital to facilitate with vaccination by covering for the cost of all its employee could improve coverage.

Other options could include setting time for vaccination at work spaces as part of employee wellness programs as was observed in Brazil and Turkey [17,18]. Continuous medical education could be of help, especially for HCWs with vaccine hesitancy who have an obligation to protect themselves and their patients as well as advocating vaccination for their patients. Additionally, promoting a supportive organizational culture that vaccines are mandatory and prioritizes employee’s health and well-being, along with integrating vaccination initiatives within comprehensive employee wellness programs [19]. Laboratory staff in this study were twice as likely not to vaccinate as compared to doctors a finding observed in other developing countries like Afghanistan, Haiti, Malawi, Nepal, and Senegal [20]. In Ethiopia, Laboratory technologists were 12.5 times less likely to complete HBV vaccination series as compared to doctors [21]. In Nigeria, nurses and health attendants were also less likely to vaccinate when compared to doctors [22]. In this study we observed that HCWs with 10 years or less of employment were less likely to vaccinate. Some reasons explained by other studies include limited exposure to occupational health policies, lower prioritization of personal health interventions in the early careers, or lack of access to vaccination programs targeted at newer employees [19,23,24]. Similarly, a study in Gauteng province in South Africa indicated that shorter employment duration correlated with lower vaccination rates [25]. These findings highlight the importance of hospital management to have targeted interventions to improve vaccination rates among newly recruited employees.

Evaluation of immune response to HBV vaccine among healthcare workers helps to strategize for additional HBV vaccine booster shots as well as the level of safety of HCWs from acquiring HBV. The majority of vaccinated HCWs mounted protective levels of immunity against HBV infection with efficacy levels surmounting to the levels recommended by WHO of 90–95% [1].

However, of note is that a quarter of the HCWs had immunity from previous HBV infection. This observation highlights the existence of natural immunity among HCWs at MNH emphasizing the importance of serological screening of HCWs before vaccination to avoid unnecessary vaccination. Contrary to our study a tertiary hospital in Mwanza found the immune response to be 77.1% among participants who were fully vaccinated [6]. This study didn’t assess the reasons for the low response rate to the HBV vaccine. But a higher prevalence of HBV infection was observed, which could potentially hinder the immune response to the HBV vaccine. The presence of HBV infection may interfere with the effective mounting of the vaccine-induced immune response

The distribution of HCWs with protective immunity among vaccinated HCWs at MNH shows the complex interplay between vaccination efficacy, natural immunity, and individual immune responsiveness.

## Conclusion and recommendations

The prevalence of HBV infection among HCWs at MNH is low, with an overall low HBV vaccination rate. There is a need to aim at zero infection rate to safeguard transmission of HBV from staff to patients. Targeted strategies such as education of HCWs to limit vaccine hesitancy while mandating a requirement for vaccination for all newly employed staff in addition to setting time, and provision of the HBV vaccine to HCWs can improve vaccine uptake.

Vaccine hesitancy was one of the factors for low vaccine coverage we recommend qualitative studies to explore further on reasons for hesitancy to address the specific barriers for vaccination in a tailored approach.
